# On the Pathology of Insanity

**Published:** 1853-10-01

**Authors:** John Hitchman

**Affiliations:** Derby County Asylum, Mickleover


### ON THE PATHOLOGY OF INSANITY.

To the Editor of the Journal of Psychological Medicine.

Sir,—An interesting work, entitled, "On the Nature and Proximate Cause of Insanity," has been kindly forwarded to me by a friend—and as it professes, among other matters, to deal with certain pathological opinions of mine, which opinions must have been derived mainly from the pages of the "Psychological Journal," you will allow me, perhaps, some space in that Journal, to correct the errors which, by partial quotations, the author of the above work has promulgated respecting them.

I am unwilling that the facts upon which my opinions are based should be distorted and misrepresented; or that *opinions which I repudiate utterly* should be given to the world *as mine*, upon so high an authority as that of Dr. Davey.

At page 13 of the cited publication, the author writes, "I have already stated, on the authority both of Foville and Dr. Hitchman, that in cases of acute mania, the grey matter of the brain is found of "a most intense redness, approaching to that of erysipelas," or "injected and of a rose colour." It must be a source of regret to him, earnest in the pursuit of truth, and without the opportunity found in an establishment like Hanwell, or Colney Hatch, to collect the necessary facts for himself, to discover so very manifest a discrepancy in the reports and opinions of our most practical and scientific men. Thus, whilst Foville and Dr. Hitchman affirm that the grey matter is *injected* and of a *rose* or *scarlet* colour, Mr. Solly is found to declare the same structure to present, and in the same disorder (mania), "*a dark plum colour.*" And further, I have before me the notes of a post-mortem examination of M. A., a patient of the Hanwell Asylum in 1842, and who died there under my care, *during a paroxysm of acute mania*, in whom Dr. Conolly has reported that "the cineritious" substance was "generally very pale," but for these, and not less the foregoing contradictions to which I have had occasion to draw the attention, I hope to offer a most satisfactory explanation in the succeeding remarks; and more than this, to render the same the means of demonstrating the *boná fide* nature and seat of mental derangement in all its various phases.

No further elucidation of my opinions is given, except such extracts *as would leave the reader to believe* that I *limited* the physical causes of insanity to a roseate condition of the cerebral hemispheres. Whereas, the very contrary is the opinion which I have inculcated both in the clinical classes at Hanwell, and in my published writings. The "plum colour" observed by Solly, and the "generally very pale appearance" recorded by Dr. Conolly, are by no means "contradictory" to my views, for in the very lecture from which I suppose Dr. Davey has culled these, "contradictions," I state, that "insanity may be produced by *impaired nutrition*," as well as by "an excess of blood, or its too active circulation;" or the converse of these from various conditions of the heart, and other causes. Indeed, the object of all my labours has been to show the fallacy of those theories which would assign so complicated a malady as insanity to *one* special corporeal ailment, whether that ailment be "deficient nervous tone," which has been so ably, elegantly, and modestly enforced by Dr. Henry Monro, or be a "sanguineous erethism," as advocated by Guislain. (See No. 12 of this Journal.) While the examination of several hundred lunatics has convinced me, that one *structure* is *mainly* implicated in this malady, I am equally certain that the *pathological conditions* are most *numerous*, and that either a "roseate hue," a "plum colour," or a very "pale state of the cerebral convolutions," may be the concomitants of extensive mental disease; as, on the other hand, no physical change whatever may be detected after death, the disturbance of the cerebral structure being induced either mechanically by pressure of contiguous structures—by blood—poison, or by vital sympathy with remote organs.

As the faithful historian of what I saw, I stated that, in three cases of recent mania, the convolutions were found of a roseate hue, but *I have nowhere stated* that they *are not found of other colour* than this. On the contrary, "the burden of my song" has been, that "there will occur a *multiplicity* of *visible changes* in that structure, by any of which insanity may be caused;" and the last time I ever had the honour to address a public assembly of medical men, my words were these—"In conclusion, it may be restated, that, *with one exception*, in every case of acute idiopathic mania (*i.e.* which I had seen), the cineritious neurine of the hemispherical ganglia was found of a roseate or reddish hue, and this accords with the larger experience of Foville. In some cases there is reason to infer that this erythema may disappear in the act of dying, or immediately after death, as an inflamed conjunctiva may become blanched in the act of syncope. In dementia this structure is often pale, soft, and atrophied. *My whole object is, however, to show that insanity is not dependent upon any* special *form of disease in this structure*, but that *any extensive pathological change of this part* of the brain will be productive of mental derangement; and further, that all other physical disturbances, which produce mental disease do so by creating functional disorder, or organic change in the grey portion of the cerebral convolutions; and that thus, we may still discover *a unity of principle*, even where facts appear discrepant and contradictory."—*Transactions of P. Med. Assoc.*, p. 152.

The special views of Dr. Davey do not demand any notice from me. They have been fairly criticised in your remarks on Dr. Monro's work (Journal, p. 341), in which it is shown that the *asthenic* character of insanity has been fully recognised by Mr. Nesse Hill and Crichton; while the researches of Marshall Hall had abundantly proved that analogous *symptoms* might be produced either from an excess or defect of blood in the brain. My thanks are, however, due to Dr. Davey for associating my name, even for a passing moment, with those of Foville, Conolly, and Solly; and especially do I thank him for confirming my opinions in language so like my own as the following:—

| Davey. | Hitchman. |
|---|---|
| "There can be no doubt that the various effusions, opacities, adhesions, and vascularities, and so on, which appear on the examination of the brain and its investing membranes, in persons dying insane, must be regarded as the mere effects of the cerebral disorder, and not as its first cause."—Page 38, Op. Cit., 1853. | "I believe that before the scalpel can reveal opacity, thickening and infiltration of the membranes, or congestion, inflammation, softness, or hardness of the medullary matter, there must have been great and important changes long going on, and that necroscopic appearances ought to be regarded more as results than causes, as the effects rather than the source, of the malady."—*Lancet*, vol. ii., 1847, p. 564. |
| "In the grey matter of the brain, then, is located the proximate cause of insanity."—Page 35, Op. Cit., 1853. | "Idiotism is the result of deficient or diseased cineritious matter (existing from birth) in the convolutions of the brain; and that insanity is caused by some change, either functional or organic, in the same structure."—*Lancet*, Dec. 1847. |

"Admitting that the proximate cause of insanity consists in an irritation (or morbid sensibility) of the grey matter."—Page 36, 1853.

"Each and all of these causes act by producing functional disturbance or structural change in the vesicular neurine (grey matter) of the encephalon."—*Psychological Journal*, 1850.

"Any disease whatever in the cineritious neurine of the convolutions of the brain, whether it be strumous deterioration, inflammation, or anæmia, which involves, directly or indirectly, a large extent of this important structure, may be productive of mental derangement." — *Provincial Transactions*, 1852.

These extracts are introduced with no invidious feeling towards my friend Dr. Davey, least of all, to insinuate the fault of plagiarism against him; for of this I believe him to be wholly incapable; but for the purpose of showing that observers may *agree*, as well as differ, and especially for the purpose of removing one "of those discrepancies" which are a source of "so much regret to him" (the unhappy wight!) "who, earnest in the pursuit of truth," has never had the good fortune to live either at Colney Hatch or Hanwell.

I am,
Mr. Editor,
Yours truly,
JOHN HITCHMAN.

Derby County Asylum, Mickleover,
August 17th, 1853.

---

